# *PLoS Medicine* Series on Big Food: The Food Industry Is Ripe for Scrutiny

**DOI:** 10.1371/journal.pmed.1001246

**Published:** 2012-06-19

**Authors:** The PLoS Medicine Editors

## Abstract

The *PLoS Medicine* editors ask readers to join the debate about Big Food and introduce their new series examining the activities and influence of the food and beverage industry in the health arena.

*This article is part of the the* PLoS Medicine *series on Big Food.*

Today we launch a major new series on “Big Food” in the *PLoS Medicine* Magazine. Over three weeks beginning 19 June 2012 we will publish seven articles that examine the activities and influence of the food and beverage industry in the health arena. These articles were commissioned by the senior Magazine editor (JC) under the guidance of our series guest editors Marion Nestle of New York University and David Stuckler of Cambridge University, and together they represent a multidisciplinary approach to exploring the role in health of Big Food, which we define as the multinational food and beverage industry with huge and concentrated market power [1].

Industry in health has long fascinated *PLoS Medicine* but our focus on Big Food is new. Food, unlike tobacco and drugs, is necessary to live and is central to health and disease. And yet the big multinational food companies control what people everywhere eat, resulting in a stark and sick irony: one billion people on the planet are hungry while two billion are obese or overweight [2].

The time is ripe for *PLoS Medicine* to shine a light on Big Food. Foremost, large food and beverage companies now have an undeniably influential presence on the global health stage. Whether it's food company executives providing expertise at major conferences and high-level UN meetings (e.g., [3]) or major global health funders lecturing on what nongovernmental organizations can learn from Coca-Cola [4], the perspectives and experiences of Big Food are shaping the field of global health. At the same time that their expertise is elevated in global health debates, food companies are rebranding themselves as “nutrition companies,” offering business acumen and knowledge in food science and distribution, and asserting authority over solutions to problems not just of food production but of malnutrition, obesity, and even poverty. The legitimization of food companies as global health experts is further fueled by the growing number of private-public partnerships with public health organizations [5], ostensibly designed to foster collaborative action to improve people's health and wellbeing. And yet food companies' primary obligation is to drive profit by selling food. Why does the global health community find this acceptable and how do these conflicts of interest play out?

Indeed, while problems of obesity and associated disease are dominating discussions and debates in health around the world, there's a concomitant gulf of critical perspectives on the food industry's role and competing interests. Despite *PLoS Medicine*'s longstanding interest in the tobacco, pharmaceutical, and other industries in health, for example, we have paid relatively little attention to the activities and influence of food and beverage companies: just two articles in 2007 [6],[7] and a recent editorial on the alcohol industry [8]. Searching PubMed, only an additional seven articles examining any aspect of the food industry have been published in the major general medical journals over the past 10 years.

According to Marion Nestle, these issues have been known and discussed (though not always acted upon) within the nutrition community for decades, which makes the lack of attention in the medical literature even more disappointing. In fact, Nestle's 2002 book *Food Politics: How the Food Industry Influences Nutrition and Health* is prescient in documenting a laundry list of Big Food misdeeds that are only receiving more widespread attention now: aggressive lobbying of regulators and governments, co-opting domestic and international nutrition experts, deceptive and illegal marketing to children, tactical targeting of minorities and emerging economies, and undisclosed conflicts of interest, among others, resulting in her conclusion 10 years ago that the food industry “plays politics better than anyone” [9]. More recent evidence confirms that Big Food and Big Alcohol are mimicking (and learning from) the tactics of Big Tobacco [8],[10]–[13]. In recognition, a bold move by *Journal of Public Health Policy* discourages studies of individual eating and activity [14],[15] because, as the editors state, they “have come to believe that research studies concentrating on personal behavior and responsibility as causes of the obesity epidemic do little but offer cover to an industry seeking to downplay its own responsibility.”

The *PLoS Medicine* series on Big Food is a “sampler,” offering perspectives on select topics relevant to how the food industry operates in health. In this first week the guest editors lay out a background and three competing views of how public health professionals can respond [1], and Lori Dorfman and her colleagues [16] compare soda companies' corporate social responsibility (CSR) campaigns with those of the tobacco industry, demonstrating how CSR deftly shifts responsibility for overconsumption from corporations to individuals, forestalls regulation, and promotes brand loyalty and sales. In subsequent weeks we will publish analyses of the rapid rise of Big Food sales in developing countries, an essay on food sovereignty and who holds power over food, and two perspectives from South America and Africa on the displacement of traditional diets by the incursion of multinational food companies. We decided not to provide a forum for the industry to offer a perspective on their role in global health, since this point of view has been covered many times before [17]–[20] and fails to acknowledge their role in subverting the public health agenda, thus ignoring the deeper issues that this series aims to uncover.

While our series does include perspectives from several countries around the world (including Brazil, South Africa, the UK, and the US), our series is not as regionally diverse as would be ideal. When commissioning we had a difficult time finding authors in the developing world who had not already established links with food companies (thus disqualifying them from contributing to the series, per our Magazine competing interests policy), which might be more evidence for concerns about co-opting of international nutrition experts.

The series is not comprehensive in highlighting all the relevant issues but should signal to readers our interest in considering further original research and commentary on additional areas to do with the food industry in health, including marketing to children, litigation, regulatory efforts, the impact of agriculture systems, solutions to obesity and noncommunicable diseases, and the growth and spread of markets in emerging economies. Clearly issues of nutrition and diet are key to human health and to the health of the planet. We look forward to continuing to be part of the dialogue and invite readers to join the debate via twitter (hashtag #plosmedbigfood) and to comment on the articles, which will be published over three weeks and collected at http://www.ploscollections.org/bigfood.
